# Educational Outcomes of Adolescents Participating in Specialist Sport Programs in Low SES Areas of Western Australia: A Mixed Methods Study

**DOI:** 10.3389/fpsyg.2021.667628

**Published:** 2021-05-21

**Authors:** Eibhlish O'Hara, Craig Harms, Fadi Ma'ayah, Craig Speelman

**Affiliations:** ^1^School of Education, Edith Cowan University, Mount Lawley, WA, Australia; ^2^School of Arts and Humanities, Edith Cowan University, Joondalup, WA, Australia; ^3^School of Medical and Health Sciences, Edith Cowan University, Joondalup, WA, Australia; ^4^School of Education, Curtin University, Perth, WA, Australia

**Keywords:** Specialist Sport Programs, adolescence, academic achievement, school engagement, low SES, mixed methodology

## Abstract

Specialist Sport Programs (SSPs) are an underexamined activity that combines the best features of two different contexts for adolescent development: a sporting program and a secondary school. A mixed-methods study was conducted to determine the influence of participation in SSPs on the educational outcomes of lower secondary students in Western Australia. The results demonstrated a significant improvement in specialist students' mean grade for Mathematics over the course of a year, while their mean grade for all other subjects, and their level of engagement with school, remained stable over the same period of time. Semi-structured interviews were also conducted with key stakeholders (e.g., specialist students and their parents, as well as teachers and graduates of the SSPs). Overall, the participants felt that SSPs had a positive influence on students' engagement with school, and that this engagement had a positive impact on their academic achievement. Taken together, the results of this research suggest that there is a role for SSPs in promoting positive educational outcomes for lower secondary students attending public schools located in low SES areas.

## Introduction

Engagement with learning is one of the four main goals of adolescence, which if achieved by the age of 15 years, sets an individual up for a successful transition into adult life (Blum et al., 2014). Research examining student–school engagement demonstrates that students' engagement with school can predict their grades in core subjects (Hazel et al., 2014). Although academic achievement is only one aspect of education, it is important with regard to students' future economic and social opportunity (Department of Education and Training, 2018). Other factors that may influence adolescents' educational outcomes include the socio-economic status (SES) of the adolescent and their participation in sport.

### Socio-Economic Status (SES)

Low SES has been found to have a negative association with adolescents' educational outcomes. In comparison to their higher SES peers, students from low SES backgrounds are more likely to experience school failure (Brownell et al., 2010). In Australia, it has been reported that students from low SES backgrounds have lower levels of school attendance (Hancock et al., 2013), academic achievement that is below the minimum national standard [Australian Bureau of Statistics (ABS), 2011], and lower levels of secondary school completion (Lamb et al., 2015).

### Participation in Sport

In contrast, sport participation during secondary school has been found to have a positive association with adolescents' educational outcomes, with sport participants reporting more positive educational outcomes than non-participants (Barber et al., 2001). Systematic reviews focusing on sport and physical activity conducted within schools have concluded that, as well as having a positive influence on adolescents' motor performance and self-concept, there is a positive association between adolescents' participation in sport and physical activity and their academic achievement (Rasberry et al., 2011; Demetriou and Honer, 2012).

However, negative outcomes have also been associated with adolescents' participation in sport (Garry and Morrissey, 2000; Scanlan et al., 2005), and some parents have voiced concerns that the time spent in physical education and school sport may interfere with students' academic achievement. These parents state that in order to improve students' educational outcomes, schools should focus on academic subjects and reduce the amount of time spent in physical education (Bailey et al., 2009).

It has been acknowledged that the structure and context of the sporting activity plays an important part in the development of either positive or negative outcomes (Mahoney and Stattin, 2000; Biddle and Asare, 2011). Therefore, it is important to investigate the educational outcomes associated with more specific sport contexts. An underexamined form of school-based sport is the Specialist Sport Program (SSP).

### Specialist Sport Programs (SSPs)

An SSP is a secondary school sport program through which students specialise in one sport in place of a range of elective subjects. The aim of the SSP is to develop the students' psychomotor, tactical, and physiological capabilities while the students continue their more conventional academic education (Gross and Murphy, 1990). SSPs are similar to both extracurricular and varsity sports. With all three types of sport program, adolescents specialise in one sport and dedicate a certain amount of time to their involvement in the sport. However, SSPs are also different to extracurricular and varsity sports. Specifically, SSPs are organised and delivered to the students mostly during school time in place of other elective subjects. In comparison, varsity sports, although delivered by the school, are conducted outside school hours; and extracurricular sports are delivered out of school hours through the community.

In the Australian context, enrolment in an SSP is open to all students, including those who live outside a school's catchment area (Harriss and Cibich, 1999). However, selection into an SSP is generally based on the following criteria: a high level (or potential high level) of sporting ability, a high level of coachability, a positive attitude toward sport and school, and a good record of behaviour and school attendance (Harriss and Cibich, 1999). According to Goddard (1995), some SSPs can be very selective, while others will take any student who applies in order to maximise enrolment numbers. Schools usually allocate around 4 hours of class time per week to SSPs. In the lower secondary years (Years 7–10), this time is split evenly between practical and theoretical work; whereas in the upper secondary years (Years 11 and 12), there is roughly a 70–30% practical–theoretical split (Goddard, 1995).

As well as focusing on skill development, practical sessions aim to develop and maintain students' fitness levels and can also involve weight training and an injury prevention/management focus (Harriss and Cibich, 1999). Theoretical sessions cover topics such as biomechanics and physiology, rules and tactics, nutrition, and sport psychology; as well as time management, alternative sporting career options, and social skills development (Harriss and Cibich, 1999). Through the SSPs, it is also often possible for students to gain umpiring and coaching qualifications.

Broad claims are made suggesting the positive influence of such programs on adolescents' developmental outcomes. For example, the Department of Education in WA states that SSPs can “develop character, teach technical skills and self-discipline, and nurture a love of sport. [and]. enable children to compete at the highest levels and develop their skills as athletes both on the field and in the classroom” (Department of Education, 2018, online). However, only two studies have investigated the influence of participation in an SSP on adolescents' academic achievement.

Levacic and Jenkins (2006) used the General Certificate of Secondary School Examination (GCSE) results to compare the academic performance of schools with specialist programs (such as SSPs) to schools without specialist programs. The GCSE is a standardised measure of students' academic achievement in their final year of compulsory education. The GCSE results were used to rank order schools into league tables that distinguish between “successful” and “unsuccessful” schools. Although the effect size was modest, Levacic and Jenkins (2006) study demonstrated a significant difference in the academic performance between the school types. Specifically, there was a larger improvement over time in the GCSE results of schools with SSPs than schools with either specialist arts or language programs, or in schools without a specialist program.

Taylor (2007) also investigated the influence of specialist programs by examining the position of schools on a league table based on students' GCSE results. Students attending specialist schools were again found to have better academic achievement than those attending non-specialist schools. However, the margin of difference was less than that outlined by Levacic and Jenkins (2006). Additional analyses conducted by Taylor (2007) also revealed that the observed improvement in academic achievement over time for schools with SSPs was greater at schools with a high percentage of students from low SES backgrounds.

### The Current Study

Despite the broad claims that are made suggesting the positive influence of such programs on adolescents' developmental outcomes, and some research conducted internationally, there has been no research conducted on SSPs in Western Australian schools to support the Department of Education's assertion. With 33 SSPs in Western Australia (WA)—15 of which are in low SES areas—SSPs have the potential to influence many students, yet the impact of participation in an SSP remains largely underexamined.

Specifically, there has been no investigation of adolescents' academic achievement in relation to their participation in SSPs conducted within schools located in low SES areas of Australia. Nor has there been any investigation of students' academic achievement in relation to their participation in SSPs conducted earlier in their secondary school education. The existing studies (both conducted in the U.K.) examined students' academic achievement in relation to participation in SSPs using data from the students' final year of compulsory education. As such, the influence of SSPs on the academic performance of younger adolescents is unknown. Additionally, the existing studies analysed data at a school level, rather than at the level of the student. Although an improvement over time was observed, this was for a different cohort of students with a potential difference in level of academic achievement from their predecessors. It is therefore important to examine academic achievement in relation to SSPs at a student, rather than school, level.

Furthermore, students' engagement with school, which is considered to be an essential element for overcoming the educational disadvantage adolescents face due to their low SES (Parkville Global Advisory, 2014), has not previously been measured to determine if differences between specialist and non-specialist students exist. Additionally, as adolescence is a continual process of progress toward maturity, it is important to examine adolescents' educational outcomes over time. Therefore, it is important for research to investigate both the academic achievement and school engagement of students in relation to their participation in an SSP over time.

Thus, the purpose of the current study was to understand whether participation in an SSP can influence early adolescents' educational outcomes. Specifically, the current study sought to determine if participation in an SSP has a positive influence on the educational outcomes of adolescents attending schools located in low SES areas of WA. It was hypothesised that participation in an SSP would have a positive influence on adolescents' academic performance and that this positive influence of SSPs on adolescents' academic achievement would be due to higher levels of school engagement amongst specialist students.

## Method

The current study employed a mixed methods research design. The quantitative and qualitative strands of the current research were conducted independently and simultaneously as part of a larger study of work. Data for each strand were collected and analysed independently and have only been combined at the point of interpretation.

Both quantitative and qualitative methods have numerous strengths, but also limitations. Quantitative analyses are criticised because the information they provide is detached from its real-world context; while qualitative research is often criticised for its small sample sizes and lack of generalisability (Castro et al., 2010). However, in combination these methods can negate some of the limitations that each method experiences on its own (Castro et al., 2010).

### Sampling

Purposive sampling was used to target schools offering an SSP located in a low SES area of WA. Schools that offer SSPs were identified through the WA Department of Education's webpage and the Index of Community Socio-Educational Advantage (ICSEA) score was used to define the SES of each of these schools. The ICSEA uses a compilation of information such as the students' home address, their parents' level of education, occupation, and income, and the school's location to provide each school with a number on a scale in comparison to other schools in WA [Australian Curriculum, Assessment and Reporting Authority (ACARA), 2013]. The scores on the index range from 500 (extremely educationally disadvantaged) to 1,300 (very educationally advantaged) and the median score on the index is 1,000 (S.D. 100). For the purpose of the current study, schools with an ICSEA score below the median were classified as low SES and it was assumed that students attending schools in low SES areas would come from a low SES background. Of the 32 schools in WA with an SSP, 15 were found to be in low SES areas and seven of the schools located in low SES areas agreed to participate in the research.

### Participants

Two groups of students were involved in the current research: those involved in an SSP (specialist students), and those attending the same school but not involved in the program (comparison students). Students in Year 7 through to Year 10 (12–15 years of age) were invited to participate. To recruit students into the study, the first author went to each of the schools involved to speak to the students during their physical education classes. The purpose of this visit was to provide students with information about the study and to explain what would be required of them if they chose to participate. It also provided students with an opportunity to ask the researcher any questions about the study prior to their involvement.

For the quantitative phase of the research, students could nominate to provide data relating to their academic achievement, their engagement with school, or both (achievement and engagement). With regards to academic achievement, 93 students (comprised of 68 specialist and 25 comparison students) provided informed consent. With regard to school engagement, 73 students (comprised of 64 specialist and nine comparison students) provided informed consent. The difference in participant numbers for each outcome is due to the way the data were collected. That is, to provide data for the analysis of students' academic achievement, students simply had to provide permission for the school to release their grades to the researcher, whereas, to provide data relating to their engagement with school, students had to complete an online survey (as part of a larger research project) that combined five other scales measuring students' psychosocial development.

To be eligible to participate in the qualitative phase of this research, the participants had to meet the following criteria:

- The students must be currently involved in the SSP
- The parents must have a child who is currently involved in the SSP
- The teachers must have at least 1 year of experience as an SSP teacher, and
- The graduates of the SSP must have participated in the SSP for at least a year and completed their schooling at least a year prior to the interview.

These criteria were used to ensure the participants involved in the interviews had sufficient experience with the SSP to provide an in-depth perspective of the impact of participation in SSPs. Descriptive information relating to the 22 key stakeholders involved in the qualitative phase is presented in Table 1.

**Table 1:** The number of participants (and their gender) from each school.

**School**	**Students**	**Graduates**	**Teachers**	**Parents**
1			1 (m)	
2	4 (f)	1 (m)	1 (f)	
3	3 (m)		1 (m)	1 (m) and 2 (f)
4	4 (m)		1 (m)	
5		1 (m)	1 (f)	
6		1 (m)		

*f, female; m ,male*.

The teachers of the SSPs have relative autonomy over the program on offer at their school. As such, there are some differences between the schools involved in the current study. The main difference being the sport that is the focus of the SSP. Some of the sports that the schools involved specialised in were Soccer, Rugby, Australian Rules Football, and Netball.

### Measures

Adolescents' school grades are regularly reported to them and their parents through a grading scale (A being the best grade and E being the worst grade). This grading scale is thought to demonstrate the students' achievement in relation to a school subject. For the current study, the students' academic achievement was determined by examining their grades for English, Mathematics, Science, Society and Environment (S&E), and Health and Physical Education (HPE). The students' grades were assigned the following values: A = 5, B = 4, C = 3, D = 2, and E = 1. Thus, a higher score indicated a better grade.

The Student–School Engagement Measure (SSEM; Hazel et al., 2013) has 22 items across three factors: aspirations (4 items), productivity (12 items), and belonging (6 items). The survey uses a Likert-style scale ranging from 1 (strongly disagree) to 10 (strongly agree). Scores from all items are combined to get an overall engagement score, with higher scores indicating greater engagement. Hazel et al. (2014) reported that this scale has good reliability. Tomaszek (2020) report that the Cronbach's alpha of the SSEM ranges from 0.76 to 0.84 for the factors of engagement and is 0.89 for the overall measure of engagement.

A semi-structured format was used for all interviews. Such a format allowed the participants to discuss what they felt was important regarding the benefits and challenges associated with participation. The interview included comparative, contrast, descriptive, evaluative, and structural questions, as well as probes and prompts (Smith et al., 2009). For example, the participants were asked “Can you tell me about the SSP you are involved in?” and “Can you list all the benefits of being involved in the SSP?”. As the purpose of this study was to explore the breadth of impact that participation in an SSP can have on adolescents' educational outcomes it was important to keep the interview questions open.

## Procedure and Analysis

Approval was received from the University Human Research Ethics Committee and the WA Department of Education's Evaluation and Accountability Directorate. Schools with SSPs were identified through the WA Department of Education's webpage and the ICSEA score was used to define the SES of each of these schools.

Once all consent forms were returned, the researcher provided each school with a list of students who had given permission for their grades to be collected. Schools collated the information required and provided it to the researcher either as a hard copy or as a PDF file that was sent via email. Students' grades were collected twice (Semester 1, Year 1 and Semester 1, Year 2) to allow for a repeated measures design. As such there was a 1-year gap between the baseline and final results.

The researcher also liaised with teachers to organise a time for the students to complete the SSEM. This survey was administered online through Qualtrics as part of a larger study. As such, the adolescents required access to a computer with internet access in order to participate. The online survey also collected information such as the students' name, age, school, and whether they participated in the SSP. Schools were asked to allocate an hour for the students to complete the survey, and it was completed twice with ~1 year between baseline and follow-up. The students' grades and engagement scores were de-identified as soon as the data for each year were collated.

There were two independent variables in the current study—participation (specialist or comparison students) and time (baseline and follow-up). For academic achievement, the grades for each of the five subjects (English, Mathematics, Science, S&E, and HPE) were the dependent variables. For engagement, there were four dependent variables: aspirations, belonging, productivity, and overall engagement.

A mixed repeated measures analysis of variance (ANOVA) was planned for each of the dependent variables with students' participation type as the between-groups factor. Students' scores were to be analysed across two time conditions: baseline and follow-up. However, due to a discrepancy in sample sizes (there were substantially more specialist students than comparison students), a decision was made to focus solely on the specialist students' academic performance and engagement with school over time. As such, a dependent-samples *t*-test was conducted for each of the dependent variables. Alpha was set at 0.05 due to the exploratory nature of the research and SPSS (Version 24) was used to perform the analysis.

Interpretative Phenomenological Analysis (IPA) was used to analyse the qualitative data because the research sought to examine the perceptions of several key stakeholders of the SSPs. IPA involves a double hermeneutic process; the participant first makes sense of the experience and then the researcher makes sense of the participants' perceptions (Smith et al., 2009). IPA is also an inductive and idiographic approach. That is, during IPA, the researcher looks for patterns and themes from the raw data from which to develop a general theory of the phenomenon being investigated (Smith et al., 2009). IPA takes into consideration the perspectives of the individuals involved in the experience.

Interviews were audio recorded and then transcribed. Once transcribed, the author read through the interviews while listening to the recordings to ensure the accuracy of the transcriptions. NVivo qualitative data analysis software (Version 10) was used to organise and analyse the data. Data were de-identified to ensure confidentiality and each participant was assigned a code. Male students were assigned the letters MS and female students the letters FS. Graduates were assigned the letter G; teachers, the letter T; and parents, the letter P. Each participant was then assigned a number. For example, the first male student interviewed was coded MS1.

The guidelines for analysing data using the IPA framework are flexible and can be adapted depending on the objective of the investigation (Pietkiewicz and Smith, 2014). Three general steps of IPA used in the current study were: multiple reading and making notes, transforming notes into emerging themes, and seeking relationships and clustering themes.

There are four broad criteria used to determine the validity and quality of qualitative research and the current study attempted to meet each of them. The criteria are: sensitivity to context (includes the use of relevant literature and participants' perspectives), commitment and rigour (includes methodological competence and skill), transparency and coherence (e.g., is there a good fit between the theory and method used? Are the methods and data presentation transparent?), and impact and importance (does the research enrich our theoretical understanding and does it have a practical impact?) (Yardley, 2000). As a professional courtesy, and a means of demonstrating trustworthiness of the research process, interview transcripts were sent to participants (via email) to check if they would like to make any amendments.

Once collected, the quantitative and qualitative data were analysed independently. As a form of triangulation, the findings from both phases of the research were then examined together. This allowed the authors to see how the findings from each phase could inform and validate each other.

## Results

### Quantitative Results

The analysis demonstrated a statistically significant difference over time with regards to specialist students' mean grade for Mathematics, *t*_(62)_ = 2.072, *p* = 0.042. The specialist students' mean grade for Mathematics significantly improved from Year 1 (3.08, *SD* = 0.97) to Year 2 (3.30, *SD* = 0.96). A small effect size (*d* = 0.26) was indicated by the mean difference of 0.22 between specialist students' mean Mathematics grades for Year 1 and Year 2 (99% CI = 0.44, −0.01). There was no statistically significant difference over time with regards to specialist students' mean grade in the other four school subjects. Descriptive statistics and the results of the *t*-test on specialist students' academic performance are presented in Table 2.

**Table 2:** Descriptive statistics and *t*-test results for specialist students' academic performance.

**Variables (*n*)**	**Year**	**Mean (SD)**	**Mean difference**	**95% CI**		***t***	***df***	***p***	***d***
				**Lower**	**Upper**				
English (65)	1	3.25 (0.71)	0.10	−0.11	0.30	0.903	64	0.369	0.112
	2	3.15 (0.87)							
Mathematics (63)	1	3.08 (0.97)	−0.22	−0.44	−0.01	2.072	62	0.042^*^	0.261
	2	3.30 (0.96)							
Science (55)	1	3.44 (0.96)	0.19	−0.10	0.47	1.277	54	0.207	0.172
	2	3.25 (1.13)							
S & E (54)	1	3.17 (0.84)	−0.05	−0.29	0.18	0.476	53	0.635	0.065
	2	3.22 (0.88)							
HPE (57)	1	4.39 (0.70)	0.13	−0.10	0.34	1.123	56	0.266	0.149
	2	4.26 (0.81)							

* *Significant, p < 0.05*.

The analysis demonstrated no statistically significant difference over time with regards to specialist students' aspirations, belonging, productivity, or overall engagement. Descriptive statistics and the results of the *t*-test on specialist students' school engagement are presented in Table 3.

**Table 3:** Descriptive statistics and *t*-test results for specialist students' school engagement.

**Variables**	**Year**	**Mean (SD)**	**Mean difference**	**95% CI**		***t***	***df***	***p***	***d***
				**Lower**	**Upper**				
Aspirations	1	4.60 (1.12)	0.18	−0.23	0.60	0.872	64	0.387	0.109
	2	4.42 (1.14)							
Belonging	1	5.09 (1.25)	0.20	−0.17	0.60	1.057		0.294	0.132
	2	4.89 (1.02)							
Productivity	1	6.19 (0.92)	0.06	−0.20	0.32	0.445		0.658	0.056
	2	6.13 (1.03)							
Engagement	1	6.05 (0.80)	0.13	−0.13	0.39	1.019		0.312	0.127
	2	5.92 (0.81)							

### Qualitative Results

Analysis of the interviews revealed the positive influence of participation in an SSP for adolescents attending schools located in low SES areas of WA. The overarching theme discussed by all participants was the SSPs' ability to facilitate students' engagement with school. Specifically, the SSP was perceived to facilitate students' behavioural, cognitive, and emotional engagement with school.

#### Behavioural Engagement

The SSP students' behavioural engagement was demonstrated through their compliance with the code of conduct; a prerequisite for them to remain in the program. The code of conduct outlined the teacher's expectations for the specialist students. While some schools had specific requirements for the students, such as a minimum 90% attendance rate at school and maintenance of “acceptable” grades in all subjects, other schools provided more general guidelines outlining what was expected of students in the SSP. For example, students should “be punctual, prepared, and well-presented for all classes” and “work responsibly and diligently on all activities” in school.

With regards to attendance, Participant T3 explained:

[SSPs] definitely increase the attendance of the kids. if we have it [the SSP] Period 1 [the start of the school day] and Period 5 [the end of the school day], they're [the SSP students] attending throughout the whole day.

T3 said that this structure accounted for an improvement in students' attendance in other classes as they could not be bothered to leave school in between their SSP classes. P2 concurred, “there are a lot of kids that the only reason they're still at school is because of the program—it gives them a reason to go [to school].” With regards to their behaviour and academic achievement, MS1 explained: “I'm focused on not getting into trouble, so I won't miss any games,” while MS3 said he made more of an effort with his academic studies so as to remain in the program:

It made me think, it's going to affect your appearance in the program. it's made me think harder in maths and like. English and stuff like that so. I moved up from a C to a B in English from thinking about the program, and if I didn't think about the program, I would still have been on a C kind of thing.

#### Cognitive Engagement

As well as being behaviourally engaged with their school, the SSP students were also cognitively engaged with their education. This was apparent when the students applied effort to their education because they wanted to, not because they felt they should (Sciarra and Seirup, 2008). Although it is difficult to observe cognitive engagement among students in compulsory education, this form of engagement was evident in the statements made by the teachers. For example, T2 spoke of past students who went on to tertiary level study despite it being optional: “I've kept in touch with a lot of students. saying you know ‘now I'm at university doing teaching' or ‘I've finished a masters in something else.”'

#### Emotional Engagement

The SSP students also appeared to be emotionally engaged with their school through the SSP. This emotional engagement was evident in the positive feelings the students discussed in relation to their participation in the program and the positive relationships that they reported were developed through the program. All of the SSP students said that the program was their favourite subject at school. For example, MS5 explained that participating in the SSP was “fun. it's energetic and you just have a great time doing it.” The students' positive views were echoed by the parents, with P2 saying “[my son] really enjoyed it [the SSP] and it was a good outlet for all of his energy.” The enjoyment experienced through the SSP improved the students' feelings about school in general. For example, MS7 said: “I didn't want to come to [school] unless I got into the [SSP]”; and G3 said that attending school was “the best 5 years of my life.”

It was apparent that participation in the SSPs helped to promote the students' behavioural, cognitive, and emotional engagement, with their school. Both male and female students felt that participation in SSPs positively influenced their engagement with school. However, only male students discussed specific aspects relating to engagement, such as attendance, behaviour, and academic achievement. This is a significant finding as previous research that has demonstrated gender differences in school engagement levels has found that girls were more engaged with school than boys (Dotterer et al., 2007). The graduates, teachers, and parents also discussed these aspects of engagement.

#### Integration of Findings

The findings of the qualitative research add depth to the quantitative results. The quantitative research demonstrated a stability of the students' engagement scores over time and showed their engagement levels to be in the neutral zone. On face value this may not seem like a very positive outcome. However, all participants involved in the qualitative research indicated that the SSPs facilitated students' engagement with school. For many students, the SSP was the reason they attended school each day; furthermore, it was the reason they applied effort to their education. As such, the SSPs were seen to have a direct positive influence on students' engagement with school, which enabled an indirect positive influence on their academic performance. It is therefore thought that without the SSP, the students' engagement levels (and academic performance) would decrease, as is commonly reported in the literature.

## Discussion

The aim of this study was to investigate the effect of involvement in SSPs on the educational outcomes of adolescents attending schools in low SES areas of WA. To achieve this, quantitative and qualitative research methods were used to examine the academic achievement and school engagement of specialist students over the period of a year.

The results of the quantitative phase of research demonstrate a statistically significant difference in the specialist students' mean grade for Mathematics over time. That is, over the period of a year, the specialist students' mean grade for Mathematics improved. There was, however, no significant difference over time with regards to specialist students' mean grade in the other four school subjects.

Interpretation of specialist students' academic achievement should consider the grade that the mean score represents. The students' grade in each subject describes the “expected level that the majority of students are achieving by the end of a given year of schooling” (School Curriculum and Standards Authority, 2016, p. 2). According to the School Curriculum and Standards Authority (2016), a C grade demonstrates a satisfactory level of achievement, while a B grade demonstrates a high level of achievement.

In the first year of data collection, specialist students' mean grade for English, Mathematics, Science, and S&E was a C. That means that despite the amount of time specialist students spend in the SSP, they were still achieving, on average, a satisfactory level for English, Mathematics, Science, and S&E subjects. Furthermore, the grade a student is awarded is based upon what is expected at that particular year level, so that as students move through the year levels, the expectations placed on them increase. Although specialist students' grades did not improve over the period of a year, they maintained a satisfactory level of achievement in English, Mathematics, Science, and S&E, despite an increase in the difficulty level of the content being taught.

The results of the current study showed an improvement in the specialist students' mean grade for Mathematics, but not their other school subjects. As such, the current study only provides partial support for the results of Levacic and Jenkins (2006) and Taylor (2007). Levacic and Jenkins (2006) study demonstrated a larger improvement over time in the GCSE results of schools with SSPs than schools without specialist programs. This was confirmed by Taylor (2007) who also found that schools with a high percentage of students from low SES backgrounds had a greater improvement over time, than those with a higher percentage of students from high SES backgrounds.

Due to the lack of a non-SSP comparison group, the current study cannot claim a causal association between students' participation in an SSP and their Mathematics grades. However, the results may ease parents' concerns regarding the time spent in Physical Education classes detracting from students' academic achievement (Bailey et al., 2009).

The quantitative phase of research also demonstrated that the school engagement of specialist students remained stable over the period of a year. This was an important finding as engagement with school has previously been found to decrease in early adolescence (Brown and Larson, 2009).

Examination of the mean score for each of the engagement factors showed that the school engagement of specialist students was close to the neutral response. That is, out of a possible score of 10, specialist students' mean response ranged from 4 to 6. This, however, is not to say that the SSP did not have a positive influence on specialist students' engagement with school. Unlike previous research conducted by Brown and Larson (2009), the specialist students' level of engagement with school did not decrease. Additionally, all participants involved in the qualitative phase of the research reported that participation in the program had a positive influence on the students' engagement with school.

There are three types of engagement relevant to an examination of students' engagement with school: behavioural, cognitive, and emotional (Fredricks et al., 2004). Behavioural engagement refers to students applying effort to their education (Sciarra and Seirup, 2008); cognitive engagement refers to students applying effort to their education because they want to, not because they feel obliged to (Sciarra and Seirup, 2008); and emotional engagement refers to the affective reactions students have to their teachers, peers, and the school in general (Fredricks et al., 2004). All three types of engagement were alluded to by the participants in the current research.

The positive influence of SSPs on adolescents' engagement with school is an important result as engagement with learning is essential for overcoming the educational disadvantage adolescents face due to their low SES (Parkville Global Advisory, 2014). Students from low SES backgrounds have previously been found to have lower rates of school attendance (Hancock et al., 2013) and their academic achievement is below the minimum national standard [Australian Bureau of Statistics (ABS), 2011]. The improved outcomes of attendance, behaviour, and academic achievement for students in an SSP in a low SES area increase the students' likelihood of completing secondary school and continuing into further education, which is an important step toward breaking the cycle of disadvantage that currently exists for students from low SES backgrounds (Department of Education and Training, 2018).

### Strengths and Limitations

The key strength of this research is the comprehensive approach taken to investigate the influence of SSPs. Namely, a longitudinal design using both qualitative and quantitative methods, sampling participants from multiple schools and taking into account multiple perspectives. This study is the first study to examine the educational outcomes of Australian students in relation to their participation in an SSP. It is also the first study worldwide to examine both the academic achievement and the engagement levels of low SES adolescents involved in SSPs. This research will therefore serve as a base for future studies of adolescent development in relation to participation in youth sport and school-based programs.

One of the limitations of this research is the lack of a comparison group of students. Despite the author's best efforts to recruit both specialist and non-specialist students, there were not enough non-specialist students who provided informed consent to participate in the study. Consequently, it is difficult to confirm the influence of the SSP on the specialist students' academic achievement and school engagement.

Another limitation is the possibility of self-selection bias. Although all schools with an SSP located in low SES areas of W.A. were invited to participate in the research, only seven schools agreed to do so. It is possible that only those schools in which the SSP teacher was proactive and proud of the program's accomplishments agreed to be involved in the research. There may be other schools with SSPs that have vastly different results, and the participation of only a limited number of schools in the research may highlight a lack of accountability for these programs.

## Conclusion

The purpose of this investigation was to explore the influence of participation in SSPs on the educational outcomes of students attending schools located in low SES areas of WA. Although it is difficult to confirm the influence of the SSP on the specialist students' educational outcomes, the research is strengthened by the mixed methods design that allowed for an exploration of the perspectives of multiple key stakeholders. Furthermore, the research sampled participants from seven different schools, thereby increasing the generalisability of the results because the SSPs at the schools involved focused on different sports, under the leadership of different teaching staff. As such, the results of this research make a significant contribution to the literature.

Although there is room for improvement with regard to specialist students' engagement with school, the results of the current study demonstrate that specialist students are making satisfactory academic progress despite the amount of time spent in the SSP. This is important because engagement with learning is one of the main goals of early adolescence (Blum et al., 2014). Overall, the findings of the present study point to the positive influence participation in an SSP can have for adolescents attending schools located in low SES areas.

## Data Availability Statement

The datasets presented in this article are not readily available because only the authors have ethics approval to access the dataset. Requests to access the datasets should be directed to Eibhlish O'Hara, e.ohara@ecu.edu.au.

## Ethics Statement

The studies involving human participants were reviewed and approved by Edith Cowan University Human Research Ethics Committee. Written informed consent to participate in this study was provided by the participants' legal guardian/next of kin.

## Author Contributions

EO'H, CH, and FM contributed to the conception and design of the research. EO'H was responsible for data collection, analysis and interpretation, and wrote the first draft of the article. CH, FM, and CS supervised the project. All authors provided feedback and helped to shape the research and manuscript.

## Conflict of Interest

The authors declare that the research was conducted in the absence of any commercial or financial relationships that could be construed as a potential conflict of interest.
